# Organic Dinitrates: Electrolyte Additives That Increase the Energy Densities of Lithium/Graphite Fluoride Batteries

**DOI:** 10.3390/nano15100758

**Published:** 2025-05-18

**Authors:** Junwei Xiao, Lingchen Kong, Yong Wang, Ziyue Zhao, Yu Li, Wei Feng

**Affiliations:** School of Materials Science and Engineering, Tianjin University, Tianjin 300354, China; jwxiao@tju.edu.cn (J.X.); konglingchen@pku.edu.cn (L.K.); wy2022@tju.edu.cn (Y.W.); zyzhao07@tju.edu.cn (Z.Z.)

**Keywords:** fluorinated graphite, micro-morphological design, nitrate ester-based compounds, fluoroalkyl chains, high energy

## Abstract

Li/graphite fluoride (Li/CF*_x_*) batteries display the highest energy densities among those of commercially available primary Li batteries but fail to satisfy the high-performance requirements of advanced applications. To address this drawback, two liquid organic dinitrates, namely, 1,4-butanediol dinitrate (BDE) and 2,2,3,3-tetrafluoro-1,4-butanediol dinitrate (TBD), were employed as high-energy energetic materials, and they were highly compatible with the electrolytes of Li/CF*_x_* batteries. The use of Super P electrodes confirmed that the reduction reaction mechanisms of both nitrate ester-based compounds delivered considerable specific capacities, associated with discharge potentials matching that of the Li/CF*_x_* battery. When considering the combined mass of the electrolyte and cathode as the active material, the overall energy densities of the Li/CF*_x_* batteries increased by 25.3% (TBD) and 20.8% (BDE), reaching 1005.50 and 969.1 Wh/kg, respectively. The superior performance of TBD was due to the synergistic effects of the high electronegativities and levels of steric hindrance of the F atoms. Moreover, the nanocrystal LiF particles generated by TBD induced crack formation within the fluorinated graphite, increasing the lithium-ion accessible surface area and enhancing its utilization efficiency. These combined factors enhanced the reactivity of TBD and facilitated its involvement in electrochemical reactions, thus improving the capacity of the battery. The developed strategy enables the facile, cost-effective enhancement of the capacities of Li/CF*_x_* batteries, paving the way for their practical use in energy-demanding devices.

## 1. Introduction

The numerous advantages of primary Li batteries, including their high energy densities, wide operating temperature ranges, and extended shelf lives, render them promising components of power sources [1,2]. Among commercial primary Li batteries, Li/graphite fluoride (Li/CF*_x_*) batteries exhibit the highest theoretical energy densities and exceptionally wide operating temperature ranges [3,4,5], thus holding promise for use in applications requiring high capacities and energy densities. These batteries are widely used in diverse fields, e.g., in military, aerospace, medical, and civil equipment [2,5,6]. Since the commercialization of Li/CF*_x_* batteries in the 1970s [7], substantial progress has been reported in developing the corresponding materials and understanding the related reaction mechanisms. To date, diverse CF*_x_* materials, such as fluorinated graphene, nanotubes, carbon fibers and nanoparticles, fullerene C_60_, and hard carbon, have been used in fabricating Li/CF*_x_* batteries. The performance of such a battery can be enhanced by designing fluorocarbon structures [8,9], adjusting the F/C ratio [10], and modifying the morphology of the CF*_x_* cathode [2,11,12].

However, as Li/CF*_x_* batteries cannot satisfy the increasing performance demands of modern applications, their overall performances require improvement. Although research regarding Li/CF*_x_* batteries largely focuses on CF*_x_* materials, strategies for enhancing the energy densities of these materials remain underexplored. Despite the various nanostructuring strategies examined as conventional solutions [13,14,15,16], the inherently low tap densities of such engineered nanostructures substantially hinder their practical application, and optimizing other battery components is, thus, a good option. Electrolyte optimization is particularly critical [17], as Li^+^ solvation affects the dynamics of electrolyte discharge [18], which, in turn, affects energy density. Electrolyte additives are easier to implement than purposeful electrolyte designs [19]. Yang et al. developed high-performance battery systems by incorporating ethylene sulfite additives in electrolytes to enhance their capacity while replacing conventional graphite with fluorinated graphite nanosheets exhibiting a high F/C ratio. This work demonstrated that electrolyte additives can synergize effectively with material design strategies to achieve superior battery performance [20]. Ma et al. systematically investigated the intricate reaction mechanisms of vinyl sulfite during discharge using density functional theory (DFT) calculations and experimental methods. Their work comprehensively evaluated the influences of variations in the vinyl sulfite concentration of the electrolyte on the electrochemical performance [21]. Lai et al. enhanced CF*_x_* cathode conversion utilizing a propylene carbonate (PC) + 1,2-dimethoxyethane (DME) electrolyte with a high dielectricity and moderate polarity, enabling efficient heterolytic C–F bond cleavage. Coupled with the ethylene sulfite additive forming a stable solid electrolyte interphase on the Li anode, this approach yielded a specific capacity of 1027.50 mAh/g and an energy density that was 21.89% higher than those of conventional systems [22]. Gao et al. prepared a novel class of redox-active perfluoroalkanes by engineering a stronger yet polarizable bonding environment via the incorporation of conjugated systems (e.g., aromatic groups). This strategy successfully yielded multifunctional fluoroalkyl-aromatic compounds with exceptional electrochemical activities [23]. Yang et al. developed high-performance battery systems by incorporating ethylene sulfite (ES) additives in electrolytes to enhance their capacity while replacing conventional graphite with fluorinated graphite nanosheets exhibiting a high F/C ratio. This work demonstrated that electrolyte additives can synergize effectively with material design strategies to achieve superior battery performance.

Owing to their high energy densities and multi-electron redox activities, nitrates (i.e., compounds featuring –O–NO_2_ groups) are promising electroactive materials and charge storage media [24,25,26,27]. Chen et al. designed a nitroaromatic cathode material, wherein each nitro group underwent a six-electron redox reaction, notably increasing the specific capacity and energy density of the corresponding battery [28]. Liu et al. reported a novel class of nitroaromatic compounds featuring two two-electron-accepting nitro groups and serving as high-energy multi-electron organic cathode materials [25]. The large-scale application of these materials in electrodes is challenging, primarily because of their solubilities in conventional electrolytes, but their deployment as electrolyte additives presents a promising alternative. Moreover, conventional nitrates pose safety risks because of their explosive natures, which highlights the need for further design strategies enabling the safe use of these high-energy organic molecules.

Here, we used two organic nitrates, namely, 1,4-butanediol dinitrate (BDE) and 2,2,3,3-tetrafluoro-1,4-butanediol dinitrate (TBD), and their incorporation into conventionally formulated electrolytes (0.1 M LiClO_4_ in PC/DME (1:1 in volume) effectively enhanced the energy densities of Li/CF*_x_* batteries. Mechanistic studies revealed that these additives functioned as electrochemically active materials at the reduced CF*_x_* cathode, participating in interfacial redox reactions and improving the overall electrochemical performance. The electron-rich –O–NO_2_ groups within these additives were involved in reduction during discharge, which markedly increased the specific capacity and overall energy density. Introducing the strongly electron-withdrawing F atoms into the alkyl chain of BDE (to obtain TBD) reduced the lowest unoccupied molecular orbital (LUMO) energy of the additive and, thus, lowered the redox potentials of the –O–NO_2_ groups. The synergistic effect between the high electronegativity and steric hindrance of fluorine atoms rendered TBD more reactive than BDE while suppressing subsequent free radical formation to enhance chemical stability. When these two nitro compounds were employed as additives in Li/CF*_x_* electrolyte systems, they could continue to deliver capacity via reduction reactions after the discharge of fluorinated graphite (FG) was completed, thus enhancing the overall energy densities of the batteries. The generated nanocrystalline particles facilitated Li⁺ deposition/stripping behavior, thereby optimizing interfacial characteristics while effectively suppressing Li dendrite growth, consequently enhancing commercial-grade stability.

## 2. Materials and Methods

### 2.1. Materials

The synthesis of TBD: A 250 mL three-neck flask was charged with concentrated H_2_SO_4_ (98%; 40 mL), dichloromethane (DCM; 5.6 mL), and HNO_3_ (67%; 0.2 mL) under continuous stirring. Tetrafluorobutane-1,4-diol (3.24 g; 20 mmol) was finely pulverized and gradually introduced into the reaction system. The mixture was cooled to −15 °C using an ice–salt bath and supplemented dropwise with HNO_3_ (6.0 mL) over 40 min using a pressure-equalizing dropping funnel. Subsequently, DCM (2.0 mL) was added, and the mixture was warmed to ambient temperature (25 ± 2 °C) and magnetically stirred for 24 h. The resulting mixture was slowly poured into an ice–water slurry (120 mL). The organic phase was washed with a saturated aqueous NaHCO_3_ solution, dried over anhydrous MgSO_4_, and concentrated under reduced pressure using a rotary evaporator to obtain TBD as a viscous pale-yellow [21] liquid (2.42 g; 9.6 mmol; 48% yield).

The synthesis of BDE: A 250 mL three-neck flask was charged with concentrated H_2_SO_4_ (98%; 40 mL), DCM (10 mL), and HNO_3_ (67%; 3 mL) under continuous mechanical stirring. Butane-1,4-diol (3.24 g; 20 mmol) was pulverized and introduced portion-wise into the reaction mixture. The system was cooled to −15 °C using an ice–salt bath and supplemented with more butane-1,4-diol (10 mL) using a pressure-equalizing funnel. Subsequently, HNO_3_ (6.0 mL) was introduced dropwise over 40 min using a calibrated dropping funnel. The mixture was then supplemented with DCM (2.0 mL), warmed to ambient temperature, maintained under vigorous agitation for 24 h, and then poured into an ice–water slurry (120 mL). The organic layer was washed with a saturated aqueous NaHCO_3_ solution, dried over anhydrous MgSO_4_, and concentrated under reduced pressure to afford BDE as a pale-yellow liquid (2.12 g; 11.8 mmol; 59% yield).

### 2.2. Characterizations and Calculations

The molecular structures of TBD and BDE were confirmed via nuclear magnetic resonance (NMR) and Fourier-transform infrared (FTIR) spectroscopy. The morphologies of the pristine and discharged cathodes were characterized using scanning electron microscopy (SEM) and transmission electron microscopy (TEM), respectively. The chemical compositions of the CF*_x_* powder and Super P-based discharged electrodes were characterized via X-ray photoelectron spectroscopy (XPS), whereas the corresponding crystal structures were analyzed using X-ray diffraction (XRD).

DFT calculations were performed using the ORCA 5.0 package (FACCTs, Cologne, Germany) [29,30] with the M06-2X functional [31]. With the 6-31+G(d,p) basis set [32,33], the geometries of the molecules were fully optimized in a homogeneous solution of PC–DME (1:1 in volume) using the conductor-like polarizable continuum model [34,35]. Based on the optimized geometries, frequency calculations were performed to verify that each geometry corresponded to a minimum on the potential energy surface.

### 2.3. Electrochemical Tests

Super P was utilized as the cathode material to evaluate the electrochemical performances of TBD and BDE. The homogeneously aqueous slurry comprising Super P (80 wt.%), sodium carboxymethyl cellulose (CMC; 10 wt.%), and styrene–butadiene rubber (SBR; 10 wt.%) was prepared and then coated onto Al foil, which was then dried in a vacuum oven at 120 °C for 12 h. The resulting electrode was then cut into discs with respective diameters and active material loadings of 12 mm and 9–11 mg. To avoid the influences of N and F atoms on the analyses of the reaction mechanisms of these dinitrate compounds, an electrolyte containing 0.1 M LiClO_4_ in PC/DME (1:1, *v*/*v*) and 5–80 vol.% of the desired additive was prepared. Coin cells were assembled in an Ar-filled glovebox (H_2_O < 0.1 ppm; O_2_ < 0.1 ppm) with Celgard 2034 and Li discs as the separators and anodes, respectively. Subsequently, 20 μL of the active electrolyte was drop-cast onto each electrode to evaluate the electrochemical performances of the dinitrate compounds under a constant current density of 0.1 mA/cm^2^. Their electrochemical performances were evaluated using a Land Electric battery-testing system (CT2001A, Wuhan Jinnuo Electronics, Wuhan, China), with a termination discharge potential of 1.5 V. Electrochemical impedance spectroscopy (EIS) results were obtained using a Zahner electrochemical workstation at an amplitude of 5 mV in the frequency range of 10 MHz to 0.1 Hz. The ionic conductivity (*σ*) of each prepared electrolyte was calculated using the following equation:

*σ* = *L*/(*R* × *A*),

where *A*, *L*, and *R*, respectively, represent the cross-sectional areas of the stainless-steel (SS) electrodes, the distance between the two SS electrodes, and the ohmic resistance of the symmetric SS||SS cell.

The compatibilities of these two dinitrate ester-based compounds with the Li/CF*_x_* battery were further evaluated using FG as the cathode. The process used to prepare the FG cathode was the same as that used to prepare the Super P electrodes, except the electrode components were FG (80 wt.%), Super P (10 wt.%), CMC (5 wt.%), and SBR (5 wt.%). The active material loadings of the FG cathode discs were 20 ± 2 mg/cm^2^. The coin cells were fabricated using the same procedure with the addition of 20 µL of electrolyte per coin cell to mimic the conditions of practical applications. The coin cells were subjected to galvanostatic discharge at 0.01 C based on the mass of FG.

## 3. Results and Discussion

**Figure 1:**
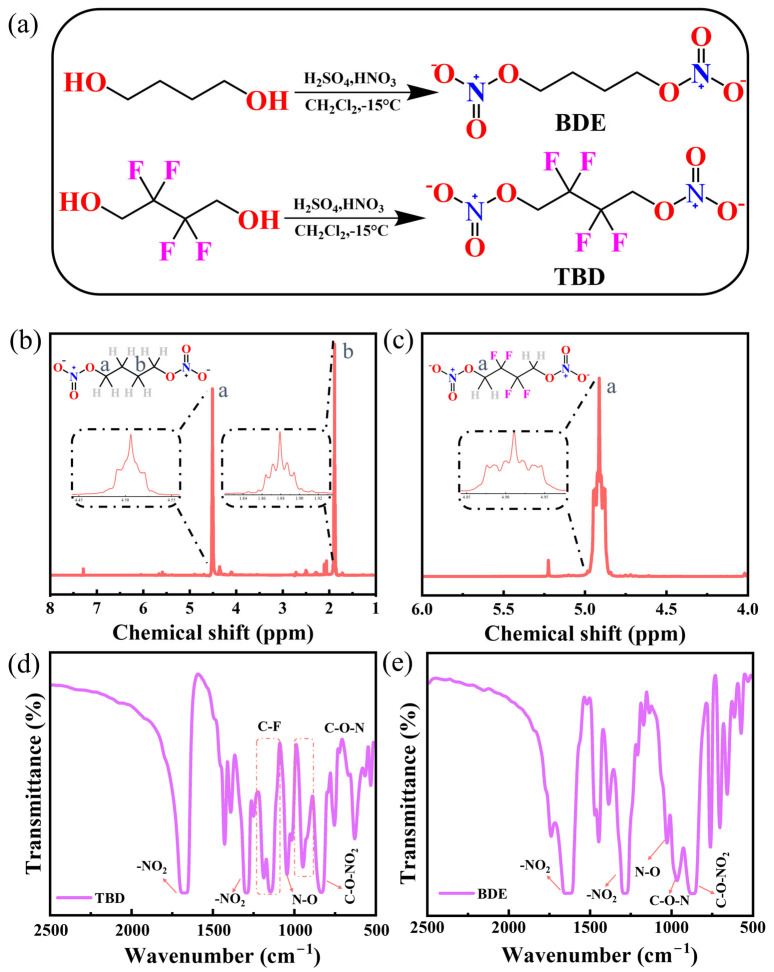
(**a**) Syntheses of BDE and TBD. (**b**,**c**) ^1^H NMR and (**d**,**e**) FTIR spectra of (**b**,**e**) BDE and (**c**,**d**) TBD.

BDE and TBD feature two –O–NO_2_ groups connected by alkyl and fluorinated alkyl chains, respectively (Figure 1a), and they have not been previously examined as active materials for use in primary batteries. The ^1^H NMR spectrum of BDE displayed peaks at 1.88 and 4.50 ppm attributable to the CH_2_CH_2_ONO_2_ and CH_2_ONO_2_ protons, respectively (Figure 1b), and that of TBD displayed a peak at 4.91 ppm attributable to the CH_2_ONO_2_ protons (Figure 1c). The FTIR spectrum of TBD (Figure 1d) featured the intense antisymmetric and symmetric stretches of the nitro groups (–NO_2_) at 1669 and 1291 cm^−1^, respectively. The FTIR spectrum of TBD also displayed the bending vibration of the N–O linkage and peak representing C–O–NO_2_ at 1043 and 834 cm^−1^, respectively. The characteristic bands associated with C–F bonding configurations with reduced binding energies were observed at 1144–1185 cm^−1^. The FTIR spectrum of BDE (Figure 1e) featured the antisymmetric (1669 cm^−1^) and symmetric (1286 cm^−1^) stretching vibrations of the nitro groups. The FTIR spectrum of BDE also displayed peaks representing the N–O bending and C–O–N vibrations at 1024 and 960 cm^−1^, respectively, with the C–O–NO_2_ moiety producing an absorption feature at 834 cm^−1^ [36].

**Figure 2:**
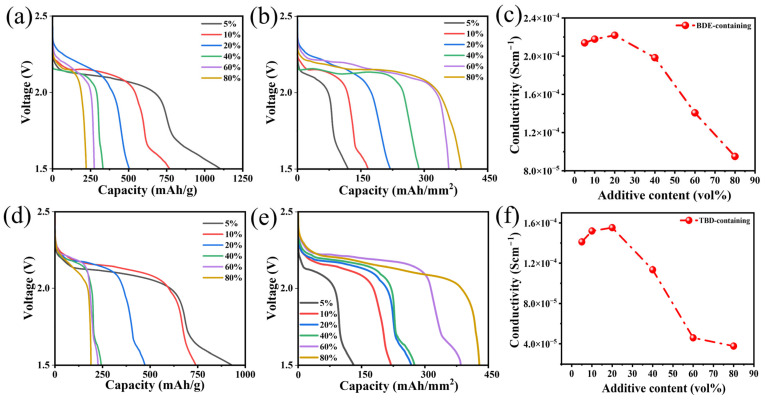
(**a**,**d**) potential–gravimetric capacity, (**b**,**e**) Potential–areal capacity, and (**c**,**f**) conductivity–additive content curves obtained using (**a**–**c**) BDE and (**d**–**f**) TBD.

The electrochemical performances of BDE and TBD were evaluated by measuring the effects of their contents on the energy output. The electrochemical performances of the Li/Super P coin cells with additive contents of 5, 10, 20, 40, 60, and 80 vol.% were evaluated at a current density of 0.1 mA/cm^2^. The corresponding discharge profiles of the electrolytes containing BDE and TBD are shown in Figure 2a and Figure 2d, respectively. At a concentration of 5 vol.%, the corresponding discharge profiles exhibited prominent plateaus at ~2.1 V and sloping plateau-like features at approximately 1.7 V. This two-stage discharge behavior suggests sequential reduction, where the plateau at 2.1 V likely corresponded to the electrochemical reduction of the –NO_2_ groups [37]. Conversely, the feature at 1.7 V was due to the adsorption of electrolyte molecules on the surfaces of the Super P nanoparticles or the further reduction of the components. Specific capacities of 930.8 and 1102.6 mAh/g were observed when using TBD and BDE, respectively. As the BDE or TBD content of the electrolyte increased, the measured specific capacity gradually decreased due to the deposition of reaction products on the electrode surface, covering the surfaces of the Super P nanoparticles and blocking further reaction. When the content of added BDE or TBD was >40%, the decreased capacity became negligible, but the areal capacity progressively increased owing to the enhanced loading of the active material (Figure 2a,d). Particularly when the content of BDE or TBD was 20 vol.%, the delivered specific capacity approached the theoretical value based on the consumption of two electrons for each nitrite [38]. Additionally, the *σ*-values of the electrolytes declined drastically after the contents of these dinitrates exceeded 40 vol.% (Figure 2c,f and Figure S1). As a result, electrolytes containing 20 vol.% BDE or TBD were used in a further analysis, unless otherwise specified.

Based on the discharge profiles of TBD and BDE, pristine electrodes and those discharged to 2.2, 2.0, and 1.5 V (Figure S2) were subjected to further characterizations. The morphologies of the deposits on the Super P electrodes were initially observed via SEM. The morphologies of the electrodes at the different discharge potentials evaluated in the electrolyte containing TBD are shown in Figure S3a–d. As the reaction progressed, the smooth spherical particles of the unreacted electrode were covered by the discharge products, and the morphology changed to a sphere-like body with grooves on the exterior. However, when using BDE (Figure S3e–h), no notable changes in the original spherical particle morphology were observed, but the gaps between the spheres were covered by the discharge products. These two distinct growth modes were due to compositional differences. The F-containing groups of TBD may be further decomposed to form LiF, whereas their absence leads to the generation of N-containing organic compounds resembling the cathode–electrolyte interphase.

**Figure 3:**
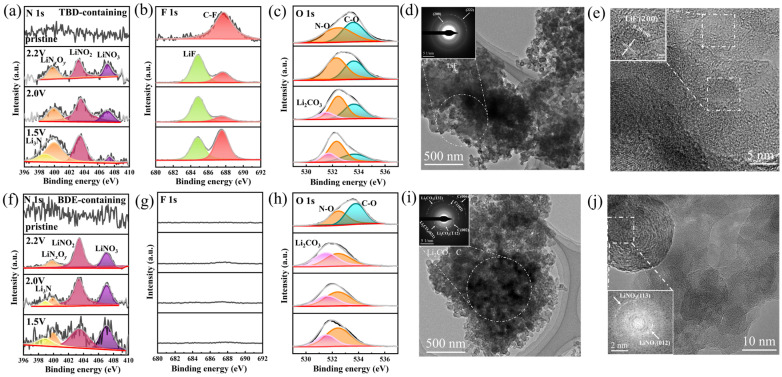
Deconvoluted (**a**,**f**) N 1s, (**b**,**g**) F 1s, and (**c**,**h**) O 1s spectra of the solid electrolyte interphases formed in the (**a**–**c**) TBD- and (**f**–**h**) BDE-containing electrolytes. High-resolution TEM images and the corresponding SAED patterns of the discharged electrodes from the (**d**,**e**) TBD and (**i**,**j**) BDE systems.

The XRD patterns of the electrodes discharged at the different potentials in the different electrolytes (Figure S4) only displayed the diffraction peaks of Super P, indicating the predominantly amorphous structures of the reduction products. Therefore, the reaction and product composition were subsequently analyzed using FTIR spectroscopy. The FTIR spectra of the electrodes at different discharge potentials in the electrolyte containing BDE (Figure S5a) revealed that the prominent peaks at 1669, 1286, and 832 cm^−1^ were no longer observed when discharged to 2.2 V. Additionally, the peak at 960 cm^−1^ was no longer observed when discharged to 1.5 V. This indicated the initial cleavage of –O–NO_2_, followed by the cleavage of –C–O–N in the potential range 1.5–2.0 V. In the FTIR spectra of the electrodes evaluated in the electrolyte containing TBD (Figure S5b), however, the disappearance of the peaks in the range of 1144–1185 cm^−1^ indicated C–F bond cleavage during discharge. This was in addition to the sequential disappearance of the peaks at 1669, 1046, and 834 cm^−1^, attributed to the cleavage of –O–NO_2_. Additionally, the disappearance of the peak at 948 cm^−1^ was assigned to the cleavage of –C–O–N. Furthermore, the electrodes discharged to various potentials were characterized using XPS, and their XPS (Figure S6) revealed the F-containing products deposited on them. The N 1s spectra of the electrodes discharged to different potentials in the electrolytes containing TBD (Figure 3a) and BDE (Figure 3f) featured peaks corresponding to inorganic nitrate (407.1 eV) and nitrite [39] (403.4 eV). In addition, the peaks at 399.9 and 398.9 eV were ascribed to LiN*_x_*O*_y_* and Li_3_N, respectively, which likely formed during the further reduction of the inorganic nitrate and nitrite [37,40].

The F 1s spectra of the electrodes discharged at different potentials in the electrolyte with TBD (Figure 3b) [41] displayed signals representing LiF (684.8 eV) and C–F bonds (687.6 eV), which were absent upon evaluation in the electrolyte with BDE (Figure 3g). The corresponding O 1s spectra of the electrodes discharged in both electrolytes (Figure 3c,h) revealed the formation of Li_2_CO_3_ (531.5 eV) [42], which was likely formed via PC decomposition. In summary, N-rich compounds were deposited on the surface of Super P via the cleavage of N-containing groups during discharge, and the fluoroalkyl chain within TBD simultaneously induced LiF formation. The structures of the products deposited on the Super P electrodes discharged to 1.5 V were further characterized via TEM. The amorphous Li–O–F–N–C matrix formed using the TBD-containing electrolyte (Figure 3d) and the corresponding selected-area electron diffraction (SAED) pattern (Figure 3d, inset) exhibited distinct diffraction rings corresponding to the (222) and (200) planes of LiF, with d-spacings of 0.14 and 0.20 nm, respectively. HRTEM characterization revealed lithium fluoride (LiF) nanocrystals with a measured (200) interplanar spacing of 0.20 nm (Figure 3e). This was consistent with the results of the XPS. Moreover, consistent with the XPS, the detected nitrates, with d-spacings of 0.21 and 0.36 nm for the (113) and (012) planes (Figure S7a), and carbonates, with d-spacings of 0.29 nm for their ($\bar{2}$02) planes (Figure S7b), were identified using the high-resolution TEM images. Conversely, when using the BDE-containing electrolyte, the signals of LiF were no longer observed in the corresponding SAED pattern (Figure 3i). Weak diffraction rings assigned to the (−132), (021), (−112), and (−202) planes of carbonates (corresponding to d-spacings of 0.15, 0.23, 0.26, and 0.29 nm) and mild features ascribed to the (113) and (012) planes of nitrates (corresponding to d-spacings of 0.21 and 0.36 nm) were detected (Figure 3j and Figure S8). The formed lithium nitrate nanocrystals contributed to the formation of the SEI, effectively suppressing Li dendrite growth. Their N-containing reduction products generated under lower-voltage conditions enhanced Li⁺ conductivity and optimized interfacial properties [43].

**Figure 4:**
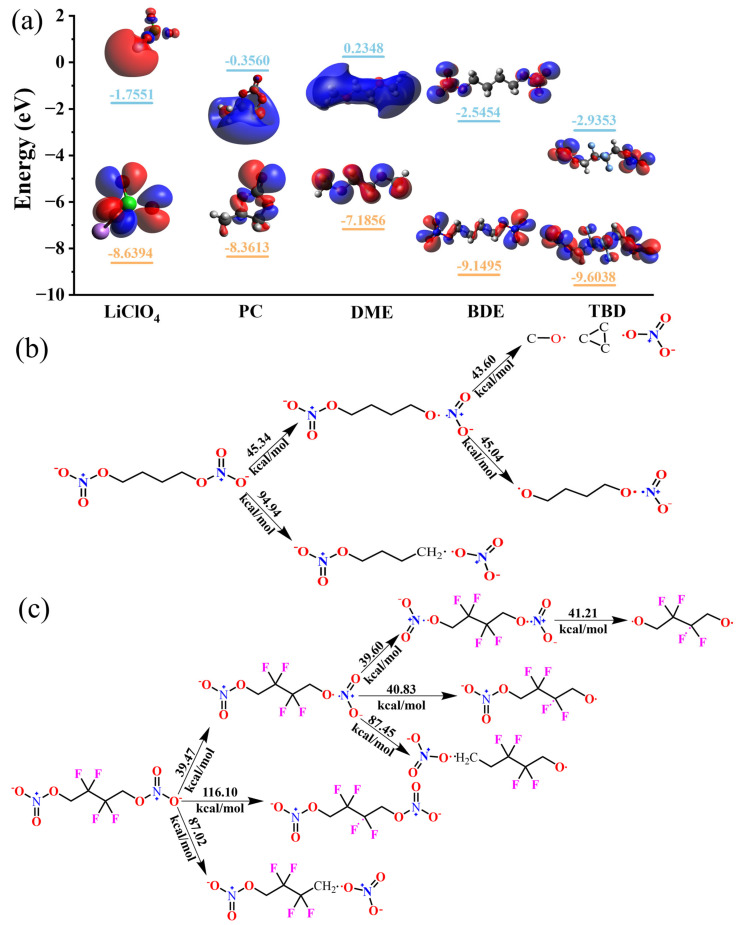
(**a**) Frontier molecular orbital energies of PC, LiClO_4_, DME, BDE, and TBD. Theoretical bond-breaking energies of (**b**) BDE and (**c**) TBD.

The reaction mechanisms were further elucidated via DFT simulations. The LUMO energy is typically used as a key factor to describe the reduction capacity of a compound. The LUMO energies of BDE, TBD, PC, DME, and LiClO_4_ were calculated using DFT, and they are shown in Figure 4a. The LUMO energies of BDE and TBD were lower than those of PC and DME, indicating that the electrolyte additives were reduced in preference to the organic solvents. TBD displayed the lowest LUMO energy, which suggests that introducing F atoms enhanced the reducibility. The LUMOs of BDE and TBD are localized at the nitro groups at both ends of the molecules, indicating the preferential reduction of these groups. Subsequently, we calculated the bond dissociation energies of BDE and TBD to determine the sequence of bond cleavage (Figure 4b,c). Upon single-electron reduction, the N–O bonds close to the main chains of BDE and TBD are cleaved first [44], which is consistent with the insights provided by the LUMO schematic. The bond dissociation energy of BDE (39.47 kcal mol^−1^) was slightly lower than that of TBD (45.34 kcal mol^−1^). This difference was ascribed to the strong electronegativities and levels of steric hindrance of the F atoms, which collectively reduced the electron density and weakened the O–NO_2_ bond, thus lowering the energy required for its cleavage. Subsequently, BDE was predicted to undergo –O–NO_2_ bond cleavage to form cyclopropane and nitrate ions (Figure S9). In contrast, TBD is more prone to N–O bond cleavage because of the increased steric hindrance due to the F atoms, which inhibits the formation of cyclic molecules. Consequently, TBD preferentially undergoes N–O bond cleavage at the terminal positions, with the bonds to the F atoms also cleaved during the process. Hence, the electrode reaction of TBD is considerably safer than that of BDE because the formation of unstable cyclic molecules during operation is prevented. Therefore, introducing fluoroalkyl chains into nitrates is an effective method of enhancing their safety in practical applications.

**Figure 5:**
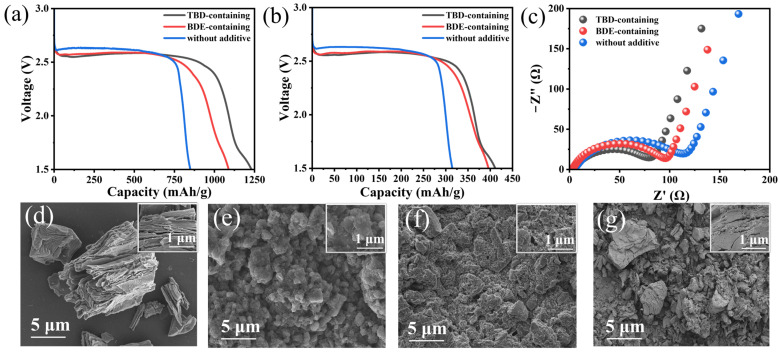
(**a**) Discharge profiles of the Li/CF*_x_* cells at 0.01 C in electrolytes with and without additives based on the mass of FG as the active substance; (**b**) corresponding discharge profiles based on the total mass of FG and the electrolyte as the active substance; (**c**) EIS of the Li/CF*_x_* cells recorded before discharge at 0.01 C. SEM images of the pristine and discharged CF*_x_* cathodes: (**d**) pristine and discharged in the (**e**) TBD- or (**f**) BDE-containing or (**g**) additive-free electrolyte. Corresponding higher-magnification images are shown as insets.

Upon considering the substantial energy densities and matched potentials of these dinitrates compared with those of the Li/CF*_x_* battery, we further investigated their compatibilities with the commercial FG cathode. The discharge profiles of the fabricated coin cells discharged at 0.01 C (1 C = 865 mA/g) exhibited plateaus at ~2.6 V (vs. Li/Li^+^), which were attributed to FG reduction (Figure 5a). Unlike the abruptly declining potential of the Li/CF*_x_* coin cell with a conventional electrolyte, the gradual decline in the discharge profile represented the involvement of dinitrates in the electrode reaction. When only FG was used as the active substance in the specific capacity calculations, the calculated specific capacities of the Li/CF*_x_* coin cells containing no additives, BDE, and TBD were 849.9, 1088.7, and 1230.6 mAh/g, respectively. The corresponding energy densities of the Li/CF_x_ coin cells containing no additives, BDE, and TBD were 2167.9, 2663.9, and 2996.0 Wh/kg, respectively. The FG cathode almost delivered the theoretical specific capacity based on its degree of fluorination (Equation (S1); Figure S10). The specific capacities of the TBD increased by 28.1% and 44.8%, respectively. When the electrolyte was considered a component of the active substance in the specific capacity calculations, the respective values of the Li/CF*_x_* coin cells with no additives, BDE, and TBD were 314.8, 396.0, and 410.9 mAh/g. The corresponding energy densities of the Li/CF*_x_* coin cells with no additives, BDE, and TBD were 1005.5, 969.1, and 802.4 Wh/kg at a current density of 0.01 C, respectively (Figure 5b). EIS was used to study the interfacial properties of pristine FG cathodes in different electrolytes. The radii of the semicircles in the high- and medium-frequency regions corresponded to the bulk (*R*_b_) and charge transfer resistances (*R*_ct_), respectively [45]. The *R*_ct_ values of the Li/CF*_x_* coin cells with the TBD- (79 Ω) and BDE-containing electrolytes (97 Ω) were marginally lower than that of the coin cell with the conventional electrolyte (115 Ω). This indicates the compatibilities of these dinitrate compounds with FG, particularly that of TBD, with fluoroalkyl chains (Figure 5c).

The morphologies and crystal structures of the pristine FG cathodes and those completely discharged in the different electrolytes were characterized via SEM and XRD. The pristine FG cathode displayed large particles (~10 µm) with typical layer-stacking structures (Figure 5d), which are unfavorable for Li^+^ transportation. After discharge, the FG cathodes were covered with nanoparticles, regardless of the electrolyte used (Figure 5e–g), which was ascribed to the LiF and nitrogen-containing inorganic compound nanocrystals generated via the conversion reaction of the FG cathode and the reduced products of the added dinitrates. Cracks were observed in the FG cathode discharged in the electrolyte with added dinitrates (Figure 5g), and they were generated due to LiF nanocrystal deposition within the FG interlayers. Nevertheless, the cracks in the conductive carbon matrix produced via the reduction of FG enlarged the reactive area and facilitated the penetration of active dinitrates. These dinitrates were reduced on the surface of the carbon substrate and then formed irregular nanoparticles comprising N-rich components on the FG cathode (Figure 5e,f). Compared with that of BDE, F-containing TBD exhibited higher compatibility with FG, as reflected by the EIS, leading to a dense, compact coating on the surface of the FG cathode. Additionally, based on the XRD patterns of the FG cathodes discharged in the different electrolytes (Figure S11), the characteristic diffraction peak representing the (001) plane of unreacted FG was no longer detected upon using the TBD-containing electrolyte. This indicates the complete reduction of the FG cathode, accompanied by the formation of amorphous reaction products [6]. Thus, TBD’s addition to the electrolyte enabled the full utilization of the capacity of FG and, thus, notably enhanced the electrochemical performances of the Li/CF*_x_* batteries.

## 4. Conclusions

The delivered capacity of a primary Li/CF*_x_* battery was notably enhanced via the addition of organic dinitrates to the electrolyte. The FG cathode, in particular, exhibited a 44.8% increase in specific capacity with the addition of 20 vol.% TBD as a functional additive. The capacity enhancement was due to the contribution of the reduction of the nitrate ester groups during discharge and the cleavage of the C–F bonds within the fluoroalkyl chains. In addition, the compatibility of TBD with the FG cathode resulted in the effective utilization of the active reagents. Theoretical calculations indicated that incorporating F atoms effectively suppressed the generation of unstable cyclic molecules via electron-withdrawing stabilization mechanisms, thus enhancing safety without compromising electrochemical performance. The N-incorporated nanocrystals deposited at the reactive interfaces formed a nitrogen-enriched SEI, effectively suppressing Li dendrite formation and enhancing its commercial-grade stability. This study provides the first experimental demonstration of nitrite compounds in the field of high-energy-density primary Li batteries and confirms that their safety can be significantly enhanced via rational molecular design strategies.

## Data Availability

The data will be made available upon reasonable request.

## Supplementary Materials

The following supporting information can be downloaded at https://www.mdpi.com/article/10.3390/nano15100758/s1. Equation (S1). Theoretical specific capacity [6]. Figure S1. Nyquist plots of the (a) 1,4-butanediol dinitrate (BDE)- and (b) 2,2,3,3-tetrafluoro-1,4-butanediol dinitrate (TBD)-containing electrolytes used to determine their ionic conductivities. Figure S2. Potential selection points in the constant-current discharge potential profile. Figure S3. Scanning electron microscopy (SEM) images of the electrodes discharged in the (a–d) TBD- and (e–h) BDE-containing electrolytes. (a,e) Pristine electrodes and those discharged to (b,f) 2.2, (c,g) 2.0, and (d,h) 1.5 V. Higher-magnification images are shown as insets. Figure S4. X-ray diffraction (XRD) patterns of the conductive carbon black-based electrodes in various discharge states obtained using the (a) TBD- and (b) BDE-containing systems. Figure S5. Fourier transform infrared spectra of the conductive carbon black-based electrodes discharged in (a) BDE and (b) TBD at different potentials. Figure S6. X-ray photoelectron survey spectra of the Super P-based cathodes in various discharge states, as acquired using the (a) BDE- and (b) TBD-containing systems. Figure S7. Transmission electron microscopy (TEM) images and the corresponding selected-area electron diffraction (SAED) patterns (insets) of the post-reaction electrodes obtained using the TBD-containing system. The images show (a) LiNO_3_ and (b) Li_2_CO_3_ formation. Figure S8. TEM images and the corresponding SAED patterns of the post-reaction electrodes obtained using the BDE-containing system. The image shows Li_2_CO_3_ formation. Figure S9. Schematic of BDE cleavage, leading to cyclopropane formation. Figure S10. (a) C 1s and (b) F 1s spectra of the CF_*x*_ powder. Figure S11. XRD patterns of the pristine and discharged CF_*x*_ cathodes (discharge was performed in electrolytes with and without additives).
