# Supplementary material for: Organic Dinitrates: Electrolyte Additives That Increase the Energy Densities of Lithium/Graphite Fluoride Batteries
Source: Nanomaterials (Basel). 2025 May 18;15(10):758. doi: 10.3390/nano15100758 (PMC12114438; doi:10.3390/nano15100758)
Supplement: Supplementary file 1 [file nanomaterials-15-00758-s001.zip › nanomaterials-3625175-supplementary.pdf]

Organic dinitrates: Electrolyte additives that increase the energy densities of lithium/graphite fluoride batteries

Characterisation of the fluorinated graphite (CF_x) powder

The F content (x, i.e. F/C ratio) of CF_x is a key parameter determining its theoretical specific capacity (Qc):

$$Q_c(\text{mAh /g}) = \frac{xF}{3.6 \times (12 + 19x)} \qquad \text{(S1)}$$

where F is Faraday's constant (96 485 C mol−1), and 3.6 is the conversion factor. X-ray photoelectron spectroscopy revealed that the employed CF_x powder exhibited an elemental composition of 50.77 at% C, 46.48 at% F, and 2.75 at% O, which corresponded to a high F/C ratio of 0.91 and Qc = 834.9 mAh/g.

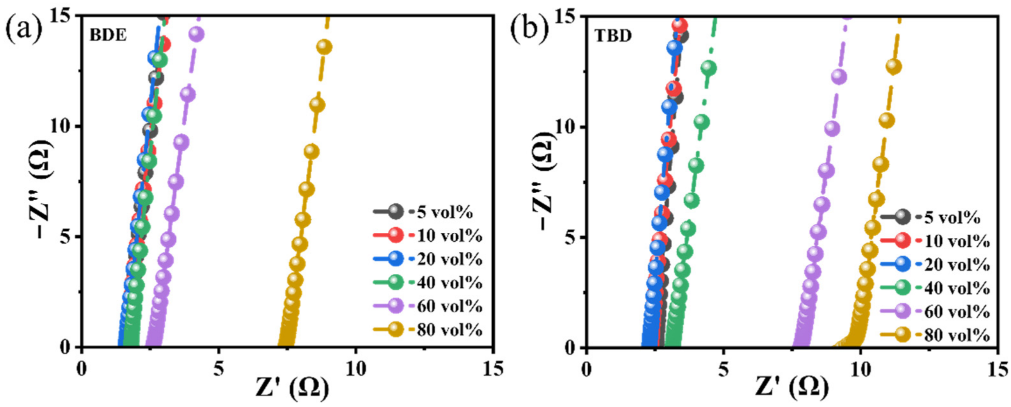

Figure S1. Nyquist plots of the (a) 1,4-butanediol dinitrate (BDE)- and (b) 2,2,3,3-tetrafluoro-1,4-butanediol dinitrate (TBD)-containing electrolytes used to determine their ionic conductivities.

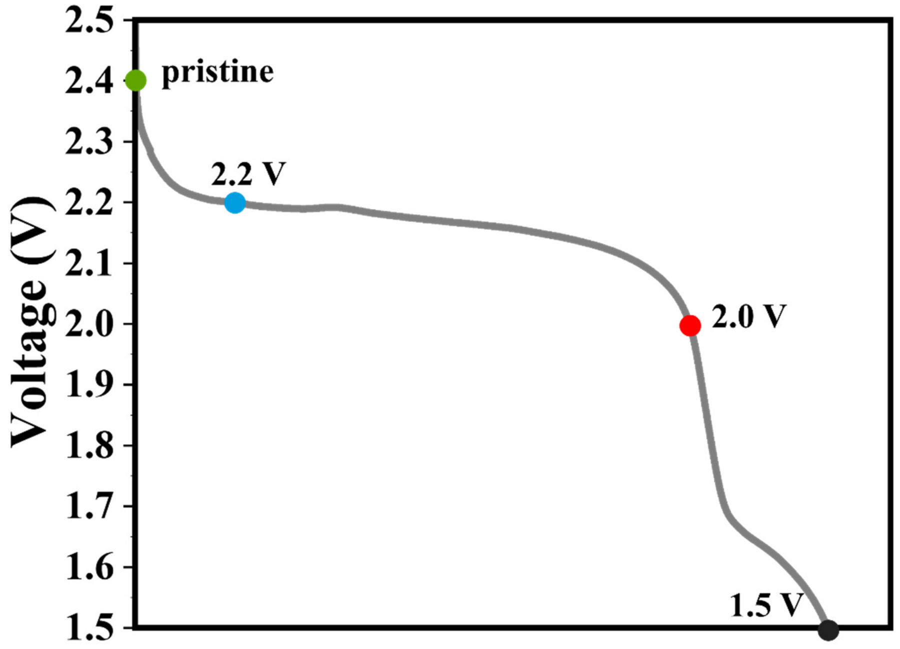

Figure S2. Potential selection points in the constant-current discharge potential profile.

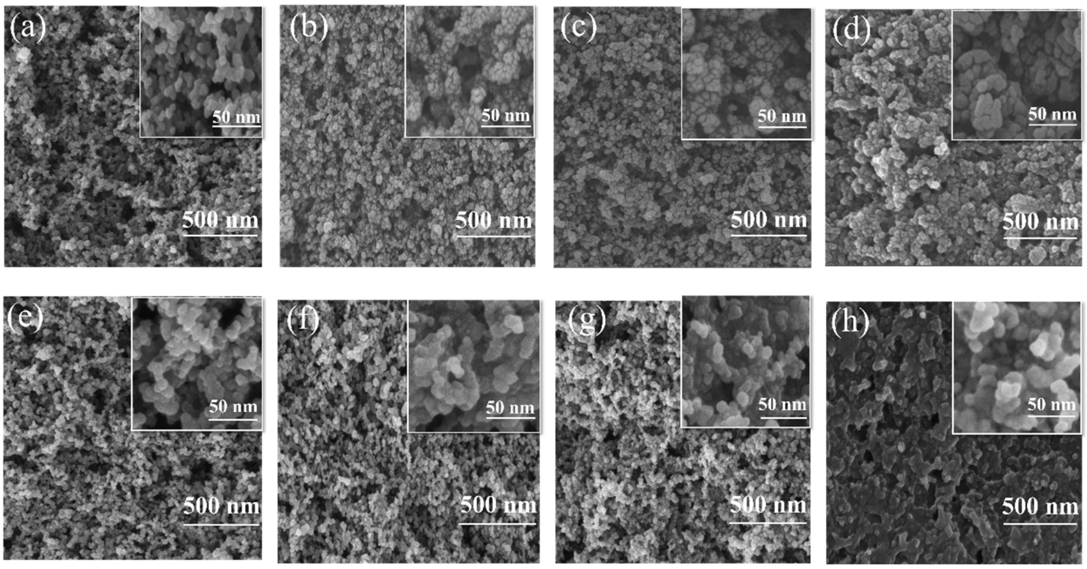

Figure S3. Scanning electron microscopy (SEM) images of the electrodes discharged in the (a–d) TBD- and (e–h) BDE-containing electrolytes. (a, e) Pristine electrodes and those discharged to (b, f) 2.2, (c, g) 2.0, and (d, h) 1.5 V. Higher-magnification images are shown as insets.

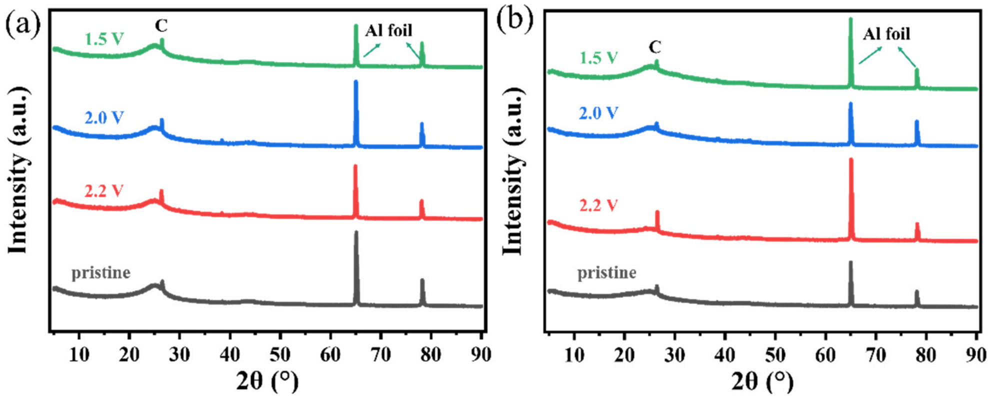

Figure S4. X-ray diffraction (XRD) patterns of the conductive carbon black-based electrodes in various discharge states obtained using the (a) TBD- and (b) BDE-containing systems.

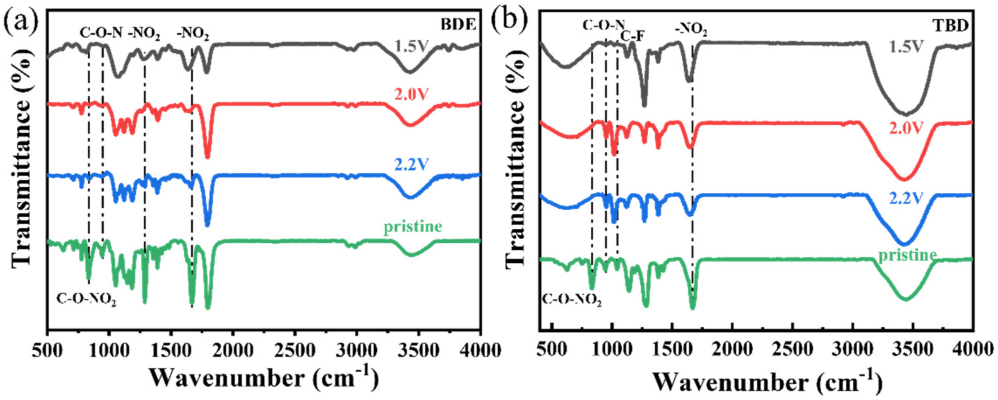

Figure S5. Fourier transform infrared spectra of the conductive carbon black-based electrodes discharged in (a) BDE and (b) TBD at different potentials.

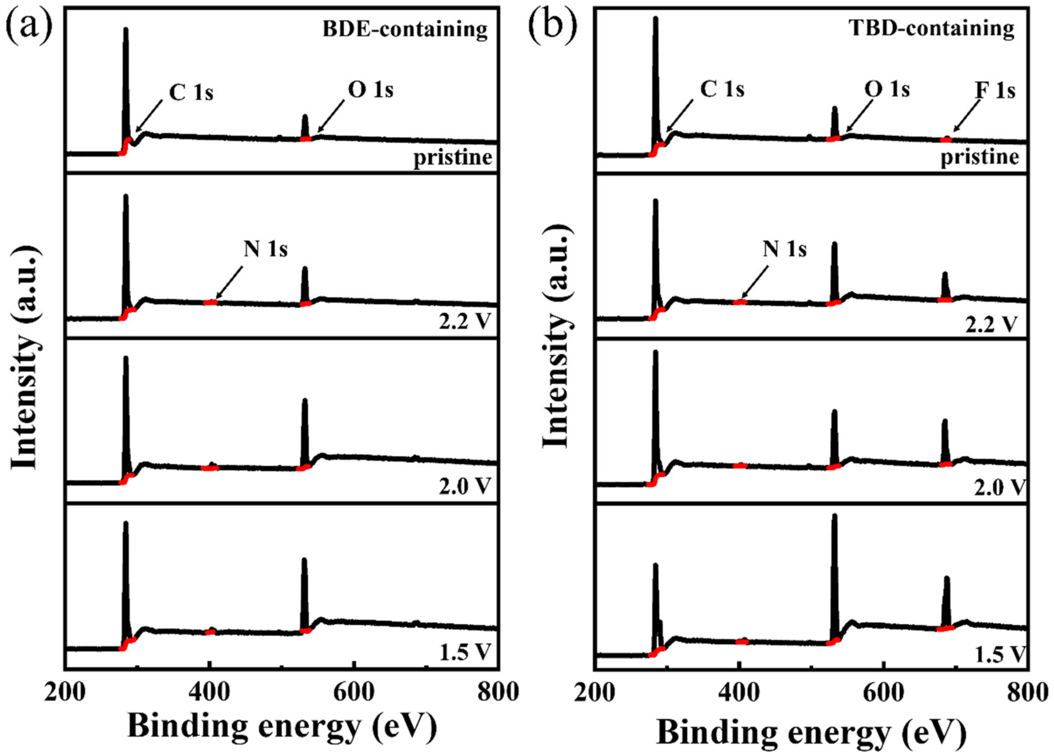

Figure S6. X-ray photoelectron survey spectra of the Super P-based cathodes in various discharge states, as acquired using the (a) BDE- and (b) TBD-containing systems.

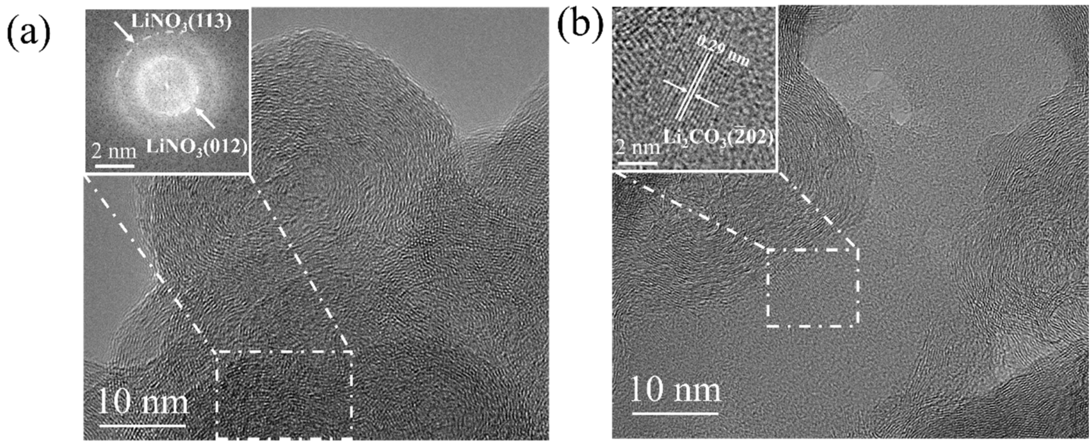

Figure S7. Transmission electron microscopy (TEM) images and the corresponding selected-area electron diffraction (SAED) patterns (insets) of the post-reaction electrodes obtained using the TBD-containing system. The images show (a) $LiNO_3$ and (b) Li_2CO_3 formation.

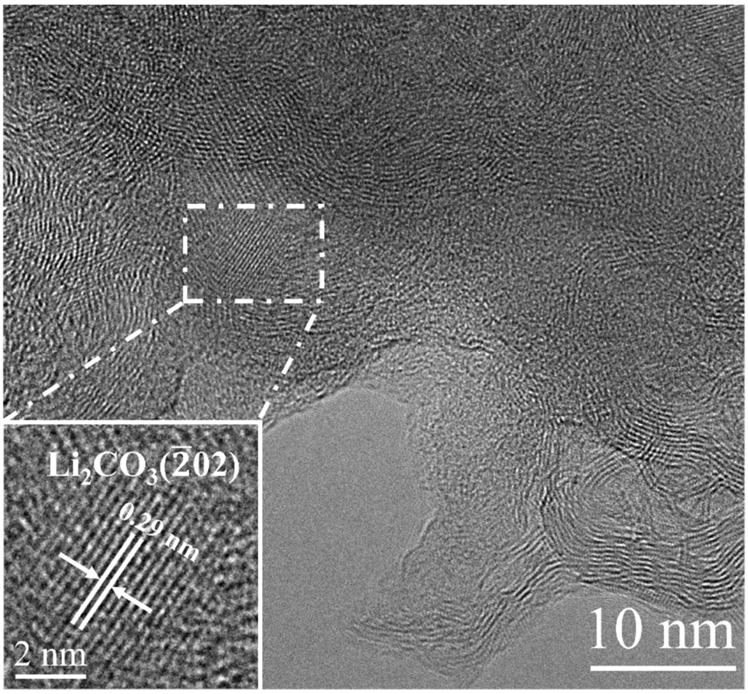

Figure S8. TEM images and the corresponding SAED patterns of the post-reaction electrodes obtained using the BDE-containing system. The image shows Li_2CO_3 formation.

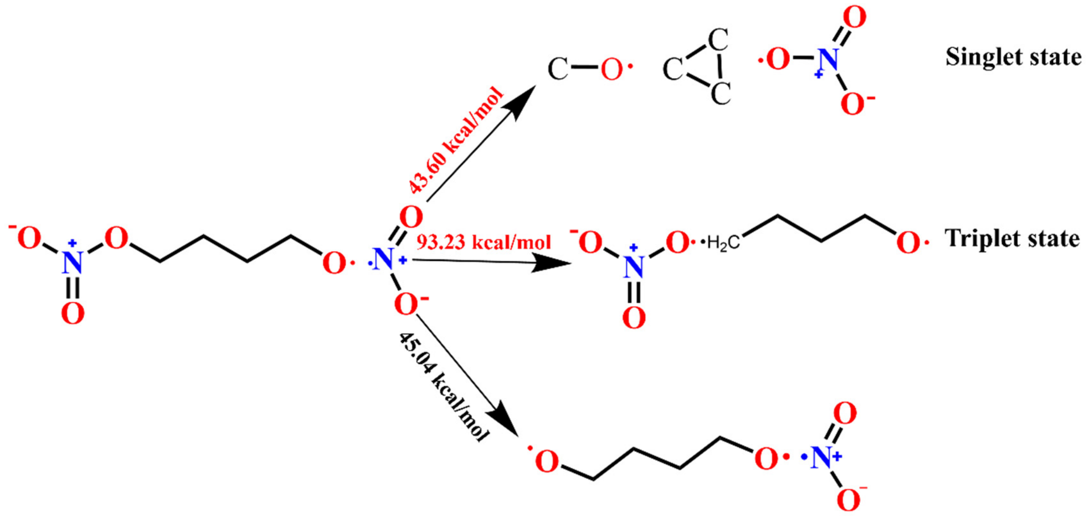

Figure S9. Schematic of BDE cleavage, leading to cyclopropane formation.

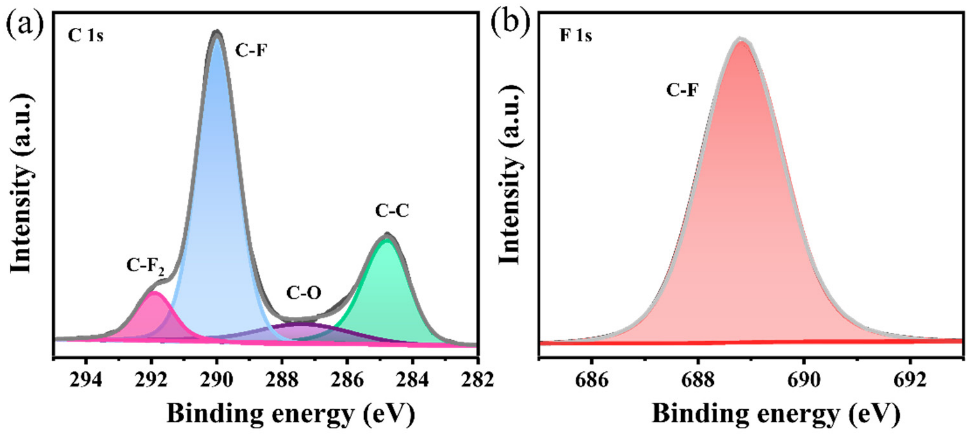

Figure S10. (a) C 1s and (b) F 1s spectra of the CF_x powder.

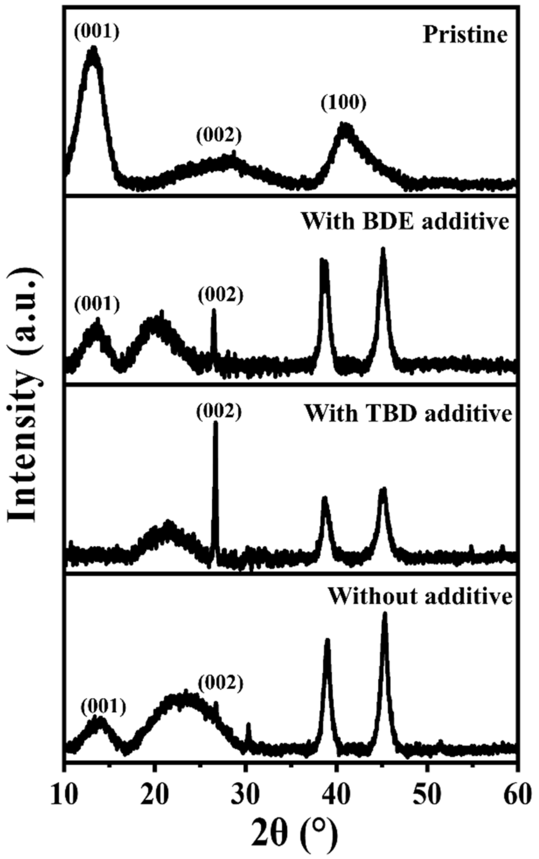

Figure S11. XRD patterns of the pristine and discharged CF_x cathodes (discharge was performed in electrolytes with and without additives).
